# Gene expression profiling of flaxseed in mouse lung tissues-modulation of toxicologically relevant genes

**DOI:** 10.1186/1472-6882-12-47

**Published:** 2012-04-20

**Authors:** Floyd Dukes, Stathis Kanterakis, James Lee, Ralph Pietrofesa, Emily S Andersen, Evguenia Arguiri, Sonia Tyagi, Louise Showe, Yassine Amrani, Melpo Christofidou-Solomidou

**Affiliations:** 1 Department of Medicine, Pulmonary, Allergy and Critical Care Division, University of Pennsylvania, 3615 Civic Center Boulevard, Abramson Research Building, Suite 1016C, Philadelphia, PA, 19104, USA; 2 The Wistar Institute, Philadelphia, PA, 19104, USA; 3 Department of Infection, Immunity and Inflammation, Leicester University, Leicester, UK

**Keywords:** Antioxidant, CAM, Flaxseed, Genomic profiling, Lignans, Lung disease, Oxidative stress

## Abstract

**Background:**

Flaxseed (FS), a nutritional supplement consisting mainly of omega-3 fatty acids and lignan phenolics has potent anti-inflammatory, anti-fibrotic and antioxidant properties. The usefulness of flaxseed as an alternative and complimentary treatment option has been known since ancient times. We have shown that dietary FS supplementation ameliorates oxidative stress and inflammation in experimental models of acute and chronic lung injury in mice resulting from diverse toxicants. The development of lung tissue damage in response to direct or indirect oxidant stress is a complex process, associated with changes in expression levels of a number of genes. We therefore postulated that flaxseed might modulate gene expression of vital signaling pathways, thus interfering with the development of tissue injury.

**Methods:**

We evaluated gene expression in lungs of flaxseed-fed (10%FS) mice under unchallenged, control conditions. We reasoned that array technology would provide a powerful tool for studying the mechanisms behind this response and aid the evaluation of dietary flaxseed intervention with a focus on toxicologically relevant molecular gene targets. Gene expression levels in lung tissues were analyzed using a large-scale array whereby 28,800 genes were evaluated.

**Results:**

3,713 genes (12.8 %) were significantly (*p* < 0.05) differentially expressed, of which 2,088 had a >1.5-fold change. Genes affected by FS include those in protective pathways such as Phase I and Phase II.

**Conclusions:**

The array studies have provided information on how FS modulates gene expression in lung and how they might be related to protective mechanisms. In addition, our study has confirmed that flaxseed is a nutritional supplement with potentially useful therapeutic applications in complementary and alternative (CAM) medicine especially in relation to treatment of lung disease.

## Background

Flaxseed (FS), a nutritional supplement known since ancient times with high contents of omega-3 fatty acids and lignans, has recently gained popularity in complementary and alternative (CAM) medicine mostly due to its benefits in cardiovascular diseases [1]. FS oil contains 52 % alpha-linolenic acid (ALA) [2], a precursor of eicosapentaenoic acid (EPA) and docosahexaenoic acid (DHA), and omega-3 fatty acids – essential fats that are both examples of polyunsaturated fatty acids. Omega-3 fatty acids help reduce inflammation and may be helpful in treating a variety of cardiovascular and autoimmune diseases [3-5]. In addition to omega-3 fatty acids (O-FA), FS also contains phenolic botanical agents called lignans. The FS lignan, secoisolariciresinol diglucoside (SDG), is metabolized in the mammalian intestine to the mammalian lignans enterodiol (ED) and enterolactone (EL), phytoestrogens demonstrating antioxidant properties [6]. The oxygen free radical scavenging properties of the FS lignans have been shown *in vitro* by either direct hydroxyl radical scavenging activity [7,8] or inhibition of lipid peroxidation [9-11]. With its additional platelet-activating-factor (PAF) antagonism [12], the lignan SDG may exert antioxidant activity by inhibiting production of reactive oxygen species (ROS) by white blood cells. The antioxidant properties of FS lignans were also verified in animal models of endotoxic shock in dogs [12], diabetes in rats [13], and in carbon tetrachloride-induced oxidative stress in rats [14]. While usefulness of the main bioactive ingredients of FS (O-FA, lignans) has been the focus of several studies, their contribution in modulation of gene expression in various tissues has never been investigated. In this work, we evaluated the effects of dietary wholegrain FS in modulating gene expression changes in lung tissues. In future studies we intend to expand our gene profiling studies to include evaluation of the FS-lignan complex (FLC).

Our group was first to investigate the role of flaxseed in acute and chronic lung injury and our findings suggested a protective role of dietary flaxseed [10,11,15-17] in murine model systems of acute and chronic lung injury. This prompted the current study, wherein the genetic profiling of flaxseed in murine lungs has been evaluated. We specifically focused on genetic changes occurring three weeks after flaxseed supplementation – the time required by lignans to achieve steady state in murine circulation as confirmed by plasma mass spectrometric analysis [15]. Mouse arrays covering 28,800 genes in the murine genome were evaluated. We first evaluated genes most up- and down-regulated in our dataset, calculated the number of statistically significant genes, and quantified our false positive rates. We then used those genes to run an aggregate pathway analysis, build gene networks according to the interactions between our significant set, and validate the results seen in the individual gene analysis. Finally, we proposed the most significant function of our test set, relative to controls. In this first reported study of genomic profiling of lung tissues in response to dietary flaxseed supplementation we focused on specific gene groups of interest shown to be relevant to acute lung injury, including antioxidant enzymes, members of the apoptotic pathway, members of the Phase I and Phase II detoxification pathways, pro-fibrogenic cytokines like TGF-beta1, and members of the cell cycle. Findings from this study will provide insight to gene-nutrient interactions thus providing scientific evidence for the usefulness of FS as a CAM modality in lung disease.

## Results

### Dietary flaxseed alters gene expression pattern in mouse lung tissues

Our group has shown that dietary FS supplementation is protective in various mouse models of pulmonary oxidative challenge including hyperoxia [15], thoracic radiation-induced injury [11,17], and ischemia/reperfusion injury [10,16]. The current study was designed to evaluate gene expression changes in lung tissues of unchallenged mice supplemented with dietary FS to elucidate the anti-inflammatory, anti-fibrotic, and anti-oxidant effects of FS. Gene expression levels from individual lung tissue samples were evaluated on separate arrays. Overall, 3,713 genes (12.9 %) were significantly (*p* <0.05) differentially expressed as a result of the diet; and of those, 2,088 (7.2 %) had >1.5-fold change.

**Figure 1:**
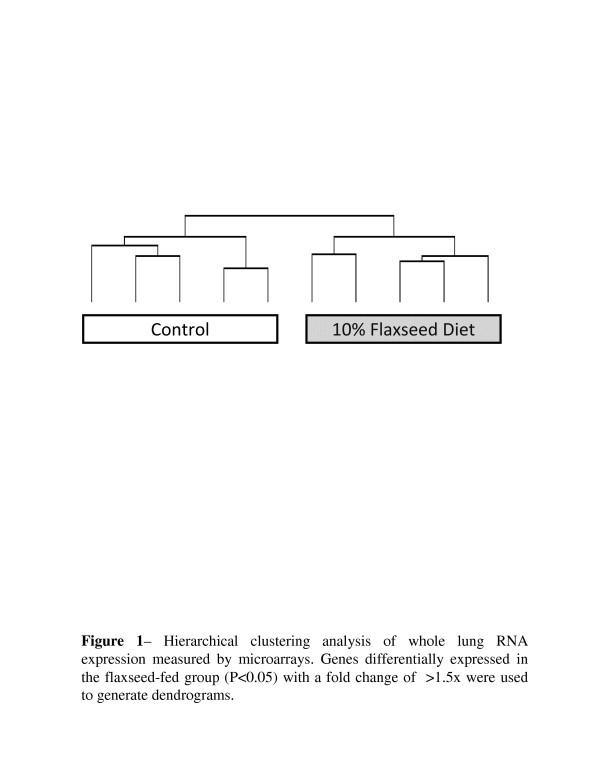
**Hierarchical clustering analysis of whole lung RNA expression measured by microarrays.**Genes differentially expressed in the FS-fed group (*p* < 0.05) with a >1.5-fold change were used to generate dendrograms.

In hierarchical cluster analysis, as shown in Figure 1, the untreated control and flaxseed-treated samples formed separate hierarchical clusters containing all of the samples from their respective groups. In principle component analysis, the two groups also formed distinct separate clusters containing all of the samples of their respective groups (data not shown).

**Figure 2:**
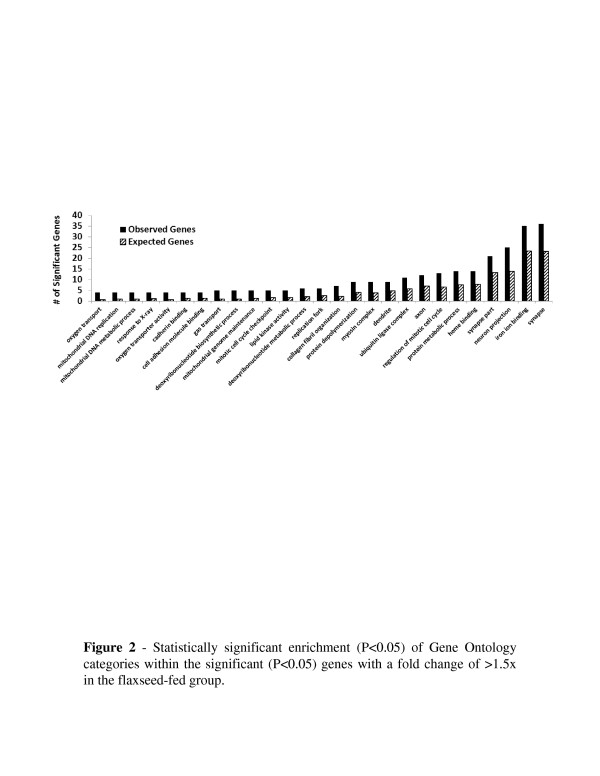
**Statistically significant enrichment (*p*** **< 0.05) of Gene Ontology categories within the significant (*p*** **< 0.05) genes with a >1.5-fold change in the FS-fed group.**

Enriched gene ontology analysis was conducted wherein significantly (*p* <0.05) overrepresented categories were identified. Within the set of genes that were significantly differentially expressed in the array, 120 ontology categories were significantly overrepresented. Figure 2 compares expected and observed representations for a selected list of ontologies. The included ontologies related to DNA synthesis, autophagy, and cell cycle progression and regulation.

Data analysis by Pathway-Express demonstrated that a number of gene pathways were significantly impacted in the FS-fed group. Table 1 lists selected pathways, including base excision repair pathway, cell cycle pathway, cytokine-cytokine receptor interaction pathway, Janus kinase-signal transducer and activator of transcription signaling (JAK-STAT) pathway, leukocyte transendothelial migration pathway, mTOR signaling pathway, phosphatidylinositol signaling pathway, and Toll-like receptor (TLR) signaling pathway. All genes from these impacted signaling pathways (many of which were down-regulated) have been provided in a separate table (see Additional file 1). Specifically, a large decrease in Rbl2 expression (−42.2 fold) suggested a down-regulation of the cell cycle pathway, as this protein is a known key regulator of activation of cell division. ATM expression was also decreased, suggesting the absence of genotoxic stress to the tissue. Cytokine-cytokine receptor interaction pathway was down-regulated with diminished expression of receptors for interleukin (IL)-2, IL-7, IL-12, IL-21, and TGF-beta.

**Table 1:** **Pathway categorization of significantly (*p*<0.05) differentially expressed genes with a 1.5-fold change in the FS-fed group**

**Pathway Name**	**Total Genes in Pathway**	**Significant Genes in Pathway**	**% Genes Significant**	**P-value**
Cell cycle	124	21	16.9 %	>0.001
Wnt signaling pathway	158	22	13.9 %	>0.001
Tight junction	136	19	14.0 %	>0.001
mTOR signaling pathway	55	11	20.0 %	>0.001
Ubiquitin mediated proteolysis	147	18	12.2 %	0.001
Toll-like receptor signaling pathway	101	14	13.9 %	0.001
Long-term depression (neurons)	80	12	15.0 %	0.001
Focal adhesion	199	21	10.6 %	0.002
Huntington’s disease	232	24	10.3 %	0.002
Leukocyte transendothelial migration	119	14	11.8 %	0.004
Long-term potentiation (neurons)	78	10	12.8 %	0.007
MAPK signaling pathway	271	24	8.9 %	0.008
DNA replication	36	6	16.7 %	0.010
Phosphatidylinositol signaling system	75	9	12.0 %	0.016
Jak-STAT signaling pathway	157	15	9.6 %	0.018
Base excision repair	53	7	13.2 %	0.022
Amyotrophic lateral sclerosis (ALS)	71	8	11.3 %	0.034
Cytokine-cytokine receptor interaction	249	20	8.0 %	0.038
Calcium signaling pathway	205	17	8.3 %	0.041
Proteasome	51	6	11.8 %	0.049

**Figure 3:**
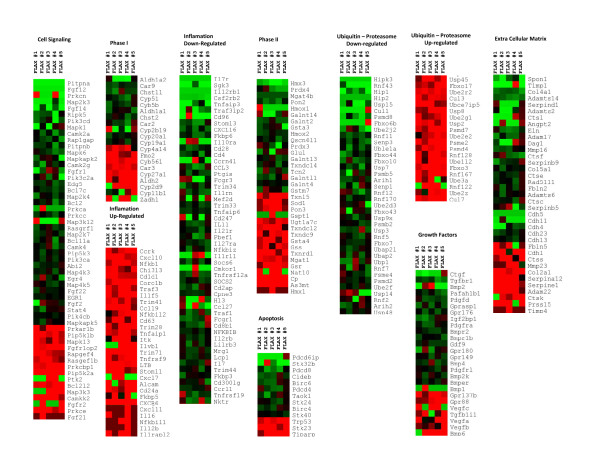
Pulmonary Gene Expression profiling of genes with >1.5-fold change in individual flax-fed mice as compared to mean of control, red indicates up-regulation, green down-regulation. Visualized in TM4 MeV.

Cluster analysis and heat map of the expression of phase I genes, phase II genes, inflammatory genes, genes involved in cell signaling and apoptosis, ubiquitin-proteasome complex genes, growth factors, and extracellular matrix genes are shown in Figure 3. All gene clusters included both up-regulated and down-regulated genes, suggesting that the impact of flaxseed lignans was complex. Various growth factors, mitogen activate protein (MAP) kinases, cytochromes P450 (CYPs), glutathione-*S*-transferases (GSTs), cadherins (CDHs), A disintegrin and metalloproteinase domain (ADAM), and chemokine (C-X-C motif) receptor (CXC) gene groups were among the set of impacted genes. Importantly, these clusters indicated that gene expression was predominantly down-regulated.

Table 1 provides other examples of important pathways in the mouse lungs that have been affected by flaxseed treatment (not all genes have been selected). FS efficiently regulated the expression of a number of genes encoding proteins that have a broad spectrum of activity. Based on its intrinsic properties, FS appeared to regulate at least five different groups of molecules essential in the regulation of (i) gene expression (transcription factors), (ii) signal transduction (signaling pathways), (iii) inflammatory responses (cytokines), (iv) cell proliferation (cell cycle regulation), and (v) cell remodeling (via cytoskeleton apparatus). These findings demonstrated that FS treatment was undoubtedly effective in driving changes of key genes in the lungs explaining, at least in part, the protective action against lung injury reported in our previous studies [10,11,15-17].

### Quantitative validation of microarray gene expression by qRT-PCR and western blot confirmation of protein levels

**Figure 4:**
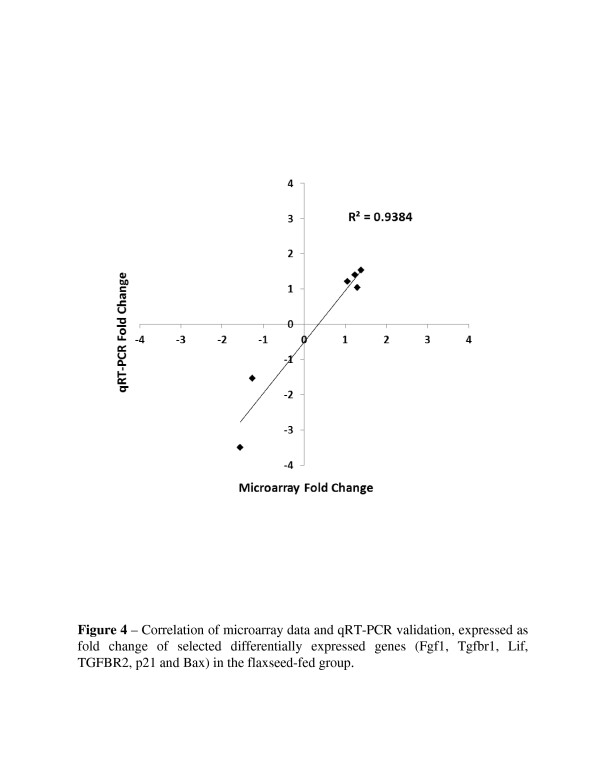
Correlation of microarray data and qRT-PCR validation, expressed as fold change of selected differentially expressed genes (Fgf1, Tgfbr1, Lif, TGFBR2, p21 and Bax) in the FS-fed group.

Reverse transcription polymerase chain reaction (RTPCR) was performed to validate the differential expression of fibroblast growth factor 1 (Fgf1), TGF-beta receptor 1 (Tgfbr1), Tgfbr2, leukemia inhibitory factor (Lif), p21, and Bcl-2–associated X protein (Bax). The changes in expression levels for these genes revealed by qRT-PCR were similar to those determined by the microarray (Figure 4).

**Figure 5:**
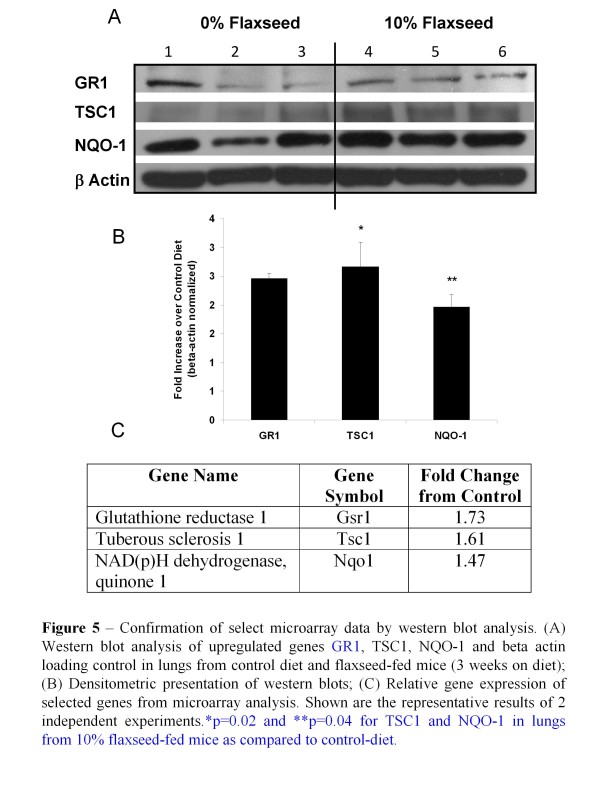
**Confirmation of select microarray data by western blot analysis. **(A) Western blot analysis of upregulated genes GR1, TSC1, NQO-1 and beta actin loading control in lungs from control diet and FS-fed mice (3 weeks on diet); (B) Densitometric presentation of western blots; (C) Relative gene expression of selected genes from microarray analysis. Shown are the representative results of 2 independent experiments. *p = 0.02 and **p = 0.04 for TSC1 and NQO-1 in lungs from 10 % flaxseed-fed mice as compared to control-diet.

Additionally, we validated some of the microarray data by Western blot analysis of select genes (Figure 5). Flaxseed is known for its antioxidant properties and thus the antioxidant and Phase II detoxification enzymes, GR1 and NQO-1, respectively were selected for protein confirmation. We also selected tuberous sclerosis protein 1 (TSC1), a multifunctional protein and member of a key pathway implicated in cell growth and metabolism, namely the Akt/TSC1-TSC2/mTOR pathway [18]. There was good correlation (R^2^ = 0.9384) between the findings of the microarray data and the Western blot.

## Discussion

Interest in the use of CAM natural products has grown significantly in recent times and FS, a botanical dietary supplement has gained significant popularity due to its antioxidant, anti-inflammatory and anticarcinogenic properties. Specifically, several studies have convincingly reported that dietary FS supplementation has a beneficial role in the management of a number of conditions including diabetes [19], lung ischemia/reperfusion injury [10], atherosclerosis [20], radiation therapy [17] and renal diseases [21] where oxidative stress is thought to be pathogenic. It is therefore important to determine the molecular mechanisms by which dietary flaxseed exerts its therapeutic action. Natural products such as FS are widely used for health purposes. Investigations about their bioactive components, their molecular and cellular targets, as well as markers of potential beneficial or harmful biological effects will provide valuable and much needed information in order to maximize their usefulness. Our study was conducted to identify natural product-induced gene regulation and/or expression changes that may identify mechanistic pathways helping to elucidate biochemical, cellular, or metabolic FS targets.

While our group has shown the functional significance of dietary FS in boosting nuclear factor erythroid 2-related factor 2 (Nrf2)-mediated protective enzyme expression in lung tissues [11], little is known in unchallenged hosts about gene expression and molecular activation changes induced by flaxseed’s anti-inflammatory, anti-fibrotic, and antioxidant properties . To date, no studies have taken a genome wide inventory of genes significantly impacted by a FS diet in unchallenged conditions. Here via gene expression analysis, we observe for the first time significant biological impacts attributed to FS.

An important outcome of this study was the demonstration that dietary FS supplementation has the potential to either positively or negatively modulate the function of a number of key regulatory proteins in the lungs thus explaining to some extent, the therapeutic value of FS reported in recent literature. Our study provides direct evidence that dietary FS leads to the expression of an array of genes that have an impact in various cellular responses that regulate cell growth and proliferation, extracellular matrix synthesis, inflammation, and oxidative stress (see Table 1). These findings will serve as the first steps to identify the gene signature by which FS exerts its therapeutic action in various experimental models of human diseases [10,11,15,17]. Of the 2,088 genes that were significantly differentially expressed with a >1.5-fold change in the FS-fed group, 1,482 (70.9 %) of those were down-regulated. Hierarchal clustering and Principle Component Analysis (data not shown) between the two groups resulted in a distinct separation between the two, indicating an overall consistency of the expression profile in individual subjects responding to the diet. In the ontology overrepresentation analysis of the significant genes (*p* <0.05 and >1.5-fold change) expressed in the FS-fed group, several ontologies were identified that related to oxygen transport, the extracellular matrix and genome maintenance processes, specifically those of the mitochondrial genome. In the context of lung disease, these processes could affect the lungs efficiency, its response to inflammation, and its response to ROS.

An important effect of FS treatment is its ability to regulate the expression of a number of molecules, including signaling molecules, which could impact the initiation and/or perpetuation of inflammatory responses. FS therapy down-regulated the expression of transcription factor ATF-2, a key target of kinases such as JNK and p38 MAPK. The concept that MAPK pathways is a natural target of FS is further supported by the fact that additional key enzymes controlling MAPK pathways were strongly down-regulated by FS including MAPK1 (also known as ERK1/2), MAPK kinase 3 (upstream of p38), and MAPK kinase 7 (upstream of JNK). As an example, MAPK kinase 3 was suppressed greater than six-fold compared to untreated controls. Although downregulation by FS of the phospho-MAPK signaling pathway in tumor tissues has been reported [22], this was the first documentation that at least in lung tissues, FS may modulate MAPK activation by downregulating expression of the upstream kinases. Importantly, a potential molecular mechanism for the protection shown by dietary FS in a mouse model of ischemia-reperfusion injury reported previously by our group [10] has been elucidated. Other studies have indeed confirmed that p38 MAPK plays a crucial role in the development of tissue injury seen in other experimental models of ischemia/reperfusion such as transplantation or myocardial infarction [23-26]. Recent studies have shown that p38 MAPK represented novel targets for the treatment of chronic lung diseases including asthma and chronic obstructive pulmonary disease (COPD) [27]. It is possible that dietary FS, by its ability to inhibit MAPK pathway activation may be a useful agent in the treatment of related lung diseases.

Acute and chronic lung injury induces an inflammatory cascade, characterized by the recruitment and activation of inflammatory immune cells in the lung [28]. Our data showed that FS modulated the expression profile of several genes encoding proteins implicated in the induction of the inflammatory pathway, as well as a decreased activation of several inflammation-related signaling pathways. Among the novel mechanisms capable of mediating the protective effect of FS in lung injury was the down-regulation of proteins called Poly ADP-ribose polymerase (PARP1 or 2). Studies using knockout mice or soluble inhibitors (INO1001, 1,7-dimethylxanthine) found that PARP1 was essential in driving the development of lung injury in response to various noxious stimuli including mechanical ventilation [29], lipopolysaccharide induced sepsis [30,31], and allergen sensitization in asthma [32-34].

The function and activation of Phase II enzymes in this experimental context left us with numerous questions about the complex nature of these compounds. Phase II enzymes play an important role in eliminating xenobiotics and their metabolites formed in Phase I reactions [35]. Genes within this group were up-regulated and down-regulated about equally, as shown in the heat map analysis (Figure 3). While genes encoding antioxidant enzymes such as GSTa3 and GSTm7 were down-regulated, other key antioxidant compounds, such as GSTa4, Txnrd1, Txndc12, Txndc9, and Sod1, were up-regulated. GSTs comprise a family of enzymes that catalyze the conjugation of glutathione to a number of endogenous and exogenous electrophilic compounds, as either membrane-bound or cytosolic compounds [35].

The gene E2F3 was up regulated in the FS diet treatment by 3.9-fold, suggesting that it may be important in the cell cycle function of lung tissue. E2F3 is thought to control cell cycle progression and proliferation in neoplastic and non-neoplastic cells [36]. Genes controlled by E2F3 seem to determine the timing of G1/S transition [37-39]. Evidence suggests that overexpression of E2F3 represents an oncogenic event during human bladder carcinogenesis and in many cases of prostate cancer [40-42].

The ubiquitin-proteasome pathways process and eliminate miss-folded or malformed proteins in the respective tissue. A highly active ubiquitin mediated proteolysis system indicates an excess of miss-formed proteins within the cell. While several genes of these pathways were up-regulated, the majority of them were down-regulated. This demonstrated that there were fewer malfunctions within cellular processing and potentially fewer cases of apoptosis. Moreover, the FS diet effectively down-regulated the majority of genes implicated in apoptosis. Down-regulation of such genes under unchallenged conditions suggested that FS might prevent apoptosis.

Leukocyte transendothelial migration is a normal part of immune surveillance in the cell. Such cell types are important to heal tissue injury and re-establish the epithelial barriers. Matrix metalloproteinases (MMPs) are extracellular endopeptidases that can function to facilitate the migration of cells by breaking down the ECM barriers, while focal adhesions are important stress fiber anchors that function in the dynamics of cell translocation [43]. Our data showed that these proteins were both up- and down-regulated, but the majority of ECM-related genes were down-regulated (Figure 3). A predominant decrease in ECM activity might mean that FS decreased the turnover and/or generation of ECM in the lung through its anti-inflammatory and anti-apoptotic activity.

## Conclusions

In conclusion, this microarray study of lung tissues from mice supplemented with a flaxseed-diet has provided unique insights into the genes that were modulated in the mouse genome secondary to the presence of flaxseed, a botanical wholegrain with potent anti-inflammatory, antioxidant, and anti-fibrotic properties. This global gene expression profile may yield further insights into the protective properties and associated cell signaling attributes of flaxseed, helping to establish this ancient wholegrain as a useful contemporary modality in complementary and alternative medicine relevant to acute and chronic pulmonary disease.

## Methods

### Animals

Female C57BL/6 mice of ages six to eight weeks were used throughout this study. All animals were cared for, handled, and housed at the Children’s Hospital of Philadelphia (CHOP) animal facility (Philadelphia, PA). All protocols were performed in accordance with National Institutes of Health guidelines and with the approval of the CHOP and the University of Pennsylvania Animal Use Committees.

### Diets and dietary treatments

The semi-purified AIN-93 G diet was used as the base diet and was supplemented with 10 % (w/w) FS as prepared by Purina Mills (TestDiet, Bloomsburg, IN). The 10 % (#56906) FS dose was selected based on published reports [44] and from our own work [10,11,15-17]. Control and experimental diets were isocaloric and equivalent in terms of the percentage of protein, carbohydrate, and fat. The Physiological Fuel Value (PFV) in all diets was kept the same, namely at 3.85 Kcal/g.

While the flaxseed seeds were stored at −80°C, the formulated chow pellets were stored at 4°C and checked regularly for oxidative degradation. Specifically, peroxide content analysis was performed at the North Dakota State University (NDSU, Fargo, North Dakota). Analysis of our diets yielded values ranging from 0.71-2.1 meq/kg reflecting negligible oxidation considering that for most food products, values of 20 meq/Kg peroxide content are considered acceptable,. Additionally, to avoid potential degradation during an experimental procedure, the diets in the cage receptacles were changed completely on a weekly basis. Whole ground yellow FS (Lot# 1012338) was kindly provided by Dr. James Hammond, (NDSU) and the North Dakota Flaxseed Council. Mice were kept on the respective diets for 3 weeks prior tissue harvest as described previously [15].

### RNA isolation, amplification, and hybridization

After the mice were sacrificed, the lungs were immediately placed in 4 M guanidine isothiocyanate, 0.5 % N-laurylsarcosine, 25 mM sodium citrate, and 0.1 M ß-mercaptoethanol solution and homogenized. Total lung RNA as described previously [45] was isolated using a modified one-step method of acid guanidinium-thiocyanate phenol-chloroform extraction [46], followed by removal of contaminating genomic DNA by DNase I treatment (Roche Molecular Biochemicals, Indianapolis, IN). Only RNA with a 260/280 ratio of > 1.7 was used.

To check for genomic DNA contamination, 2 μg of total RNA was used as a template in a PCR reaction with the primers for intronic sequences of the mouse PECAM-1 gene. No visible PCR product in total RNA sample was detected after 35 cycles, together with a positive control using as low as 500 pg of genomic DNA as a template in the PCR reaction. 0.5 μg RNA target was labeled with ^33^P, 3,000–5,000 Ci/mM using reverse transcriptase. Hybridization was in 2.5 ml Micro-Hyb (Research Genetics) at 42°C for 18 h. The first wash was terminated at 0.5x saline-sodium citrate (SSC)/ 1 % Sodium dodecyl sulfate (SDS). Filters were then exposed to a PhosphorImager screen for 4 days, scanned at 50-μm resolution on a Storm PhosphorImager, and visualized using ImageQuant (Molecular Dynamics). Filters were then further washed with 0.1x SSC/ 0.5 % SDS, exposed to a PhosphorImager screen for 7 days, scanned and analyzed.

### cDNA arrays

The cDNA filter arrays were purchased from The Wistar Institute Genomics facility (Philadelphia, PA). Three 2.5x7.5–cm nylon filters, MA-07, -10, and −11, carrying a total of 28,800 probes for individual genes were used. Specifically, MA-07 contains the first two thirds of the National Institute of Aging (NIA) 15,000 clone Mouse Developmental Library. cDNA libraries of origin were created from pre- and peri-implantation mouse embryos. MA-10 contains the remaining 5,000 genes from the NIA developmental clone set plus a set equivalent to the immunogene clone set included on MA-02 and 2,100 genes from “BMAP” clone set from Research Genetics (Carlsbad, CA). MA-11 contains Research Genetics (Invitrogen) plates 51–79: 6,079 cDNA clones from NIA mouse 7.4 K cDNA clone set, 665 selected Immunogenes and 5 Leishmania genes. These mouse arrays were used to analyze the 5 samples coming from mice fed for 3 weeks with a 10 % FS diet and 5 samples from mice on control diets. The 10 samples were hybridized as a single batch on sequentially printed arrays. All arrays used in this work were printed from the same PCR preparations.

### Array analysis

The data for each array were analyzed with ArrayVision (Imaging Research Inc., Piscataway, NJ.), using the median pixel for each spot and local background correction. Expression values for each array were normalized by the background-corrected signal median spot of the array and transformed to corresponding Z-scores for clustering. Quantile normalization was used to make the overall distribution of values for each array identical (while preserving the overall distribution of the values). It consists of two steps: i) Create a mapping between ranks and values. For rank 1 find the *n* values, one per array that are the smallest value on the array, and save their average. Similarly to rank 1, for rank 2, the second smallest values and on up to the *n* largest values (one per array) was saved and averaged; ii) For each array, we replaced the actual values with these averages [47]. The normalized and raw data from all mouse arrays used for this study was uploaded in Gene Expression Omnibus, under the following platform accession numbers: MA07: GPL2900, MA10: GPL2918 and MA11: GPL2921.

### Western blotting

Mice were fed control (0 %) or treatment (10 % FS) for 3 weeks as for genomic studies. Lungs were harvested for immunoblot analysis which was performed on whole lung homogenates as previously described [10]. Primary antibodies used included Glutathione Reductase 1 (Gr1) (Abcam, Cambridge, UK), NAD(P)H quinone oxidoreductase-1 (NQO-1) (Novus Biologicals, Littleton, CO), Tuberus sclerosis 1 (TSC1) (Cell Signaling, Danvers, MA) and Beta Actin (Sigma, St Louis, MO). Densitometry of Western blots with β-actin normalization of expression was performed using Gel-Pro Analyzer (version 6.0; MediaCybernetics, Silver Spring, MD).

### Quantitative RT-PCR: validation of selected genes

To validate the gene expression differences measured by microarray analysis, six selected genes were assessed with quantitative real-time PCR (qRT-PCR) analysis. As shown in Figure 4 the expression fold change differences of both up-regulated and down-regulated genes measured by qRTPCR were consistent with those measured by microarray analysis. Since dietary flaxseed has anti-apoptotic, anti-fibrotic and cell cycle regulating properties, we chose to evaluate genes relevant to these aforementioned processes. Genes: Fgf1 (AK084481), Tgfbr1 (BQ551162), Lif (BG079564), TGFBR2 (BG085088), p21 (AI852492) and Bax (AI323521) were chosen. Two micrograms of total RNA were reverse transcribed to cDNA using Oligo(dT)_15_ primer (Promega, Madison, WI) and powerscript reverse transcriptase (Clontech). Synthesized cDNA was then submitted to real-time PCR using either the LightCycler System (Roche Molecular Biochemicals) as previously described [11] or the SmartCycler System (Cephied, Sunnyvale, CA). The amount of cDNA was normalized using ß-actin levels. A minimum of three samples from control-diet lungs and flaxseed-fed mice were pooled and analyzed in quadruplicate. The relative expression level based on cycle number was compared between groups.

### Pathway analysis

To identify pathways modulated in the flaxseed-fed group, an analysis of significantly (*p* <0.05) differentially expressed genes with >1.5-fold change was completed using Pathway-Express [48]. This software uses pathways present in the Kyoto Encyclopedia of Genes and Genomes (KEGG) database and calculates significance, through hypergeometric distribution testing, based on the relative changes of the contained genes.

### Hierarchical clustering analysis

Clustering of the samples by expression of statistically significant (*p* <0.05) genes with >1.5-fold change was completed using the Hierarchical Clustering method in TIGR Multi Experiment Viewer [49]. The complete linkage method was used with Euclidean distance as the distance metric.

### Gene ontology enrichment analysis

Statistically significant (*p* <0.05) genes with >1.5–fold change were analyzed for enrichment of gene ontology categories with Webgestalt [50]*.* The number of observed versus expected genes were compared for selected categories calculated to have (*p* <0.05).

### Statistics

To assess the significant differences between groups in the microarray analysis, a >1.5–fold change filter and permutation based *t*-test (*p* <0.05) were performed using the TIGR Multi Experiment Viewer [49].

## Abbreviations

ALA = Acid alpha-linolenic acid; COPD = Chronic obstructive pulmonary disease; CYP = Cytochrome P450; DHA = Docosahexaenoic acid; EPA = Eicosapentaenoic acid; ED = Enterodiol, EL, Enterolactone; ECM = Extracellular matrix; FS = Flaxseed; IL = Interleukin; KEGG = Kyoto encyclopedia of genes and genomes; LA = Linoleum acid; MMP = Metalloproteinases; NQO-1 = NADAH quinone oxidoreductase-1; NIEHS = National institute of environmental health sciences; NIA = National institute on aging; NIH = National institutes of health; PAF = Platelet-activating-factor; ROS = Reactive oxygen species; SSC = Saline-sodium citrate; SDG = Secoisolariciresinol diglucoside; SDC = Sodium dodecyl sulfate; CHOP = Children’s Hospital of Philadelphia; TSCI = Tuberus sclerosis 1.

## Competing interests

The author(s) declare that they have no competing interests.

## Authors’ Contributions

FD and SK carried out the computational part of the study and assisted in drafting the manuscript. JL carried out the animal experimentation and isolated the RNA used for the array work. RP and ESA assisted with the gene validation studies to confirm array findings. EA assisted with the tissue isolation and tissue processing. ST assisted with the western blots conducting protein validation studies to complement array findings. LS supervised the gene array work and provided the data set with gene expression changes. YA assisted with the evaluation of the data. MCS supervised the project, designed the experiment and wrote the manuscript. All authors read and approved the final manuscript.

## Pre-publication history

The pre-publication history for this paper can be accessed here:

http://www.biomedcentral.com/1472-6882/12/47/prepub

## Supplementary Material

Additional file 1 Heat map-Fold Changes. Click here for file
